# Isolation and Characterization of a Frog Virus 3 Strain from a Wood Frog (*Rana sylvatica*) in Wood Buffalo National Park

**DOI:** 10.3390/v16091411

**Published:** 2024-09-03

**Authors:** Samantha R. Logan, Sibelle Torres Vilaça, Joe-Felix Bienentreu, Danna M. Schock, David Lesbarrères, Craig R. Brunetti

**Affiliations:** 1 Department of Biology, Trent University, 1600 West Bank Dr., Peterborough, ON K9J 7B8, Canada; samanthalogan@trentu.ca; 2 Environmental Genomics, Instituto Tecnológico Vale, Rua Boaventura da Silva, 955, Belém 66055-090, PA, Brazil; 3 Department of Biology, Laurentian University, 935 Ramsey Lake Rd, Sudbury, ON P3E 2C6, Canada; 4 Palustris Environmental, Athabasca, AB T9S 1H8, Canada; 5 Environment and Climate Change Canada, 1125 Colonel By Drive, Ottawa, ON K1S 5B6, Canada

**Keywords:** *Iridoviridae*, *Ranavirus*, frog virus 3, FV3, FV3-like, whole genome sequence, amphibian, viral isolates

## Abstract

Members of the *Iridoviridae* family, genus *Ranavirus*, represent a group of globally emerging pathogens of ecological and economic importance. In 2017, an amphibian die-off of wood frogs (*Rana sylvatica*) and boreal chorus frogs (*Pseudacris maculata*) was reported in Wood Buffalo National Park, Canada. Isolation and complete genomic sequencing of the tissues of a wood frog revealed the presence of a frog virus 3 (FV3)-like isolate, *Rana sylvatica* ranavirus (RSR), with a genome size of 105,895 base pairs, 97 predicted open reading frames (ORFs) bearing sequence similarity to FV3 (99.98%) and a FV3-like isolate from a spotted salamander in Maine (SSME; 99.64%). Despite high sequence similarity, RSR had a unique genomic composition containing ORFs specific to either FV3 or SSME. In addition, RSR had a unique 13 amino acid insertion in ORF 49/50L. No differences were found in the in vitro growth kinetics of FV3, SSME, and RSR; however, genomic differences between these isolates were in non-core genes, implicated in nucleic acid metabolism and immune evasion. This study highlights the importance of viral isolation and complete genomic analysis as these not only provide information on ranavirus spatial distribution but may elucidate genomic factors contributing to host tropism and pathogenicity.

## 1. Introduction

*Iridoviridae* is a family of large (~120–200 nm), circularly permutated and terminally redundant linear double-stranded DNA (dsDNA) viruses with icosahedral symmetry [1,2]. Currently, the *Iridoviridae* family is composed of six genera that infect invertebrate hosts (*Iridovirus*, *Chloriridovirus*, and *Decapodiridovirus*) or ectothermic vertebrates (*Megalocytivirus*, *Lymphocystivirus*, and *Ranavirus*) including bony fish, amphibians, and reptiles [2]. In the *Ranavirus* genus, seven species are recognized by the International Committee on Taxonomy of Viruses (ICTV), including *Ranavirus alytes1*, *Ranavirus ambystoma1*, *Ranavirus epinephelus1*, *Ranavirus gadus1*, *Ranavirus micropterus1*, *Ranavirus perca1*, and *Ranavirus rana1*, which includes the exemplar isolate frog virus 3 (FV3) [2].

Our understanding of ranaviruses has substantially improved over the past decade; however, the determinants required to produce variance in virulence in a genetically diverse group of hosts remain elusive. Members of the *Iridoviridae* family share a set of 26 core genes with proposed roles in virus structure, replication, and virulence [3,4]. Ranavirus detection, phylogenetic analysis, and taxonomic characterization have relied on comparisons with either the highly conserved major capsid protein (MCP) [5,6,7,8,9,10,11,12,13,14,15] or a very limited set of core genes [16,17,18,19,20]. Moreover, investigations of ranaviruses isolated from wild-caught animals, including during die-offs, often place emphasis on the detection of cytopathic effects (CPEs) in cell culture, ultrastructural analysis, and histopathological evaluation [6,7,11,13,14,15,17,21,22]; they do not draw comparisons between closely related isolates through complete genome sequencing. While these approaches provide considerable information, they conceal key genomic differences among viral isolates, including those that contribute to in vivo differential host effects and evolutionary relationships among isolates [23].

For example, full genome analysis of several ranavirus isolates from amphibians across northern Canada revealed robust recombination between FV3 and common midwife toad virus (CMTV) [24], while little intraspecific variation was detected in FV3-like ranaviruses from the same region through analysis using only the MCP and vIF-2α [25]. Similarly, Morrison et al. [26] investigated FV3 and a FV3-like isolate from a spotted salamander in Maine (SSME), two isolates that share 98.79% sequence identity across their entire genomes, and found that FV3 infection resulted in greater mortality rates in *Lithobates pipiens* tadpoles when compared to SSME. It was postulated that the basis for these differences in mortality was due to mutations in non-core genes and in intergenic regions [26]. Collectively, previous studies highlight the importance of whole-genome analysis and comparisons between closely related isolates, as these may help identify key differences that contribute to both pathogenicity and tropism.

In the present study, we isolated a FV3-like ranavirus, herein designated as *Rana sylvatica* ranavirus (RSR), from a wood frog (*Rana sylvatica*) in Wood Buffalo National Park, Canada, and using FV3 and SSME as reference, completed a comparative whole-genome analysis and in vitro characterization.

## 2. Materials and Methods

### 2.1. Sample Collection

**Figure 1:**
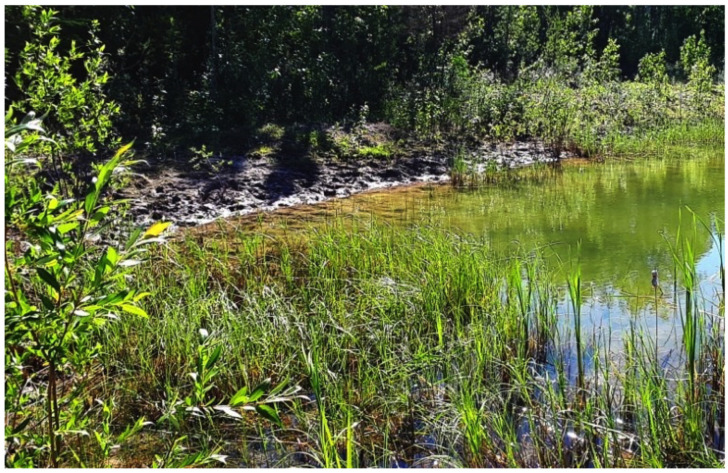
Location (60.028° N, 113.026° W) of the die-off of wood frogs (*Rana sylvatica*) and boreal chorus frogs (*Pseudacris maculata*) in Wood Buffalo National Park, Canada.

While conducting amphibian surveys in the north-eastern part of Wood Buffalo National Park, Canada (Figure 1), an amphibian die-off of wood frogs (*Rana sylvatica*) and boreal chorus frogs (*Pseudacris maculata*) was detected in late June 2017 [27]. Initial confirmation of ranavirus in amphibian tissue samples was accomplished using the protocol outlined in Leung et al. [28], which targets a diagnostic section of the major capsid protein (MCP) gene in amphibian ranaviruses. A detailed report of the initial ranavirus detection in conjunction with the associated histopathology and immunohistochemistry confirming ranavirus as the causative agent appear in Forzán et al. [27]. The ranavirus isolate examined in the current study originated from the tissues of a wood frog tadpole during this die-off and were frozen at −20 °C until virus isolation, as described below. The ecology of amphibian ranavirus dynamics in Wood Buffalo National Park, including the aforementioned die-off, are described in Bienentreu et al. [29].

### 2.2. Cell Culture and Reagents

Epithelioma papulosum cyprini cells (EPC; American Type Culture Collection, ATCC No. CRL-2872) were maintained in Leibovitz’s L-15 media (L-15; Thermo Fisher Scientific, Waltham, MA, USA) containing 2.0 mM L-glutamine supplemented with 10% fetal bovine serum (FBS; Thermo Fisher Scientific, Waltham, MA, USA), 100 U/mL penicillin (Thermo Fisher Scientific, Waltham, MA, USA), 100 μg/mL of streptomycin (Thermo Fisher Scientific, Waltham, MA, USA), and 1.5 μg/mL amphotericin B (Thermo Fisher Scientific, Waltham, MA, USA) at 20–25 °C.

### 2.3. Virus Isolation, Purification, Quantification, and DNA Extraction

A longitudinal section of a wood frog tadpole was homogenized in 200 μL of sterile 1× phosphate buffered saline (PBS) solution (pH 7.4; Thermo Fisher Scientific, Waltham, MA, USA) using a BioMasherII Closed System Micro Tissue Homogenizer (Fisher Scientific Company, Ottawa, ON, Canada). The homogenate was freeze–thawed three times to release cell-associated virus and centrifuged at 5000 rpm for 5 min to pellet cell debris. The clarified homogenate was inoculated onto a confluent monolayer of EPC cells in a 25 cm^2^ flask containing L-15 media supplemented with 1% FBS, 100 U/mL penicillin, 100 μg/mL of streptomycin, 1.5 μg/mL amphotericin B, and allowed to incubate at room temperature for 4 h. Media containing clarified homogenate was removed and cells were maintained in 1% L-15 media with regular observation for cytopathic effects (CPEs). Once 90–95% of the monolayer showed CPEs, cells were harvested, freeze–thawed three times, and centrifuged at 5000 rpm for 5 min. The newly isolated virus was serially diluted in 1% L-15 on confluent monolayers of EPC cells in a 24-well plate. Monolayers with one or two plaques were harvested, freeze–thawed three times, and re-diluted onto new monolayers; this was repeated three times to obtain an isogenic viral stock. Virus stock was quantified using a standard plaque assay. Briefly, virus stock was serially diluted in L-15 media supplemented with 1% FBS. One milliliter (1 mL) of each dilution was plated in triplicate onto confluent monolayers of EPC cells in 6-well plates and incubated at room temperature for 4 h. Inocula were removed by aspiration, and 2 mL of overlay medium (L-15 supplemented with 1% FBS, and 0.75% methylcellulose; Sigma-Aldrich, Oakville, ON, Canada) was added. Cells were incubated for 5 days, overlay medium was aspirated, and cells were stained with 1% crystal violet (Sigma-Aldrich, Oakville, ON, Canada) in 20% ethanol. The quantified viral stock was stored at −80 °C for use in DNA extraction and other downstream applications.

### 2.4. PCR Amplification, Sequencing, and Genome Assembly

DNA was extracted from infected monolayers of EPC cells using the Qiagen DNeasy Blood and Tissue Kit (Qiagen, Toronto, ON, Canada) following the manufacturer’s instructions, with resulting DNA being quantified using a NanoDrop 8000 Spectrophotometer (Thermo Fisher Scientific, Waltham, MA, USA). DNA was amplified using the 13 primers and same PCR conditions as described in Vilaça et al. [24]. Long-range PCRs for each locus were performed in 50 μL reaction cocktails consisting of 1× KAPA HiFi HotStart ReadyMix (Roche Sequencing Solutions Inc., Indianapolis, IN, USA), 0.5 μM forward and reverse primers, and 3 μL of DNA. Positive reactions were equimolarly pooled. A DNA library was prepared using the KAPA HTP library preparation kit (Roche Sequencing Solutions Inc., Indianapolis, IN, USA) using 100 ng of pooled DNA and fragmented for 10 min. The pooled library was sequenced by the TCAG DNA Sequencing Facility (Toronto, ON, Canada) on an Illumina HiSeq 2500 using 125 bp paired-end reads. Genome assembly followed the same procedure as Vilaça et al. [24]. After verifying gene order from SPAdes [30], assembly reads were mapped to the FV3 reference genome (AY548484.1) in Geneious 8.1 [31] as the genome assembly was not composed of a unique scaffold. Annotations were transferred from FV3 and SSME (KJ175144.1) using VAPiD [32].

### 2.5. Single-Step Growth Curves

Six-well plates containing 90% confluent monolayers of EPC cells in L-15 media supplemented with 1% FBS were infected with FV3 (American Type Culture Collection, ATCC No. VR-567), SSME [26], or RSR at an MOI of 5. After 4 h, inocula were removed and the monolayers were washed three times with 1× PBS (pH 7.4) and replaced with 1 mL of 1% L-15. Samples were collected at various time points post-infection and virus yields were assessed by plaque assay as described above.

### 2.6. Plaque Area

EPC cells were prepared as described for single-step growth curves. Cells were infected with FV3, SSME, or RSR at an MOI of 0.1 for 4 h. Inocula were removed, monolayers were washed three times with 1× PBS (pH 7.4), and cells were overlaid with 2 mL of 0.75% methylcellulose overlay medium. Seventy-two hours post-infection, images were taken within a 250 mm^2^ area in the center of each well using an EVOS XL Auto Imaging System (Thermo Fisher Scientific, Waltham, MA, USA) and Image J [33] was used to calculate the area of all plaques within this region. Statistical significance was assessed in GraphPad Prism 9 (GraphPad Software Incorporated, La Jolla, CA, USA) using a Kruskal–Wallis test. A *p*-value < 0.05 was considered significant, with *n* representing the number of biological replicates that were analyzed.

## 3. Results

### 3.1. General Genome Characteristics of RSR

**Figure 2:**
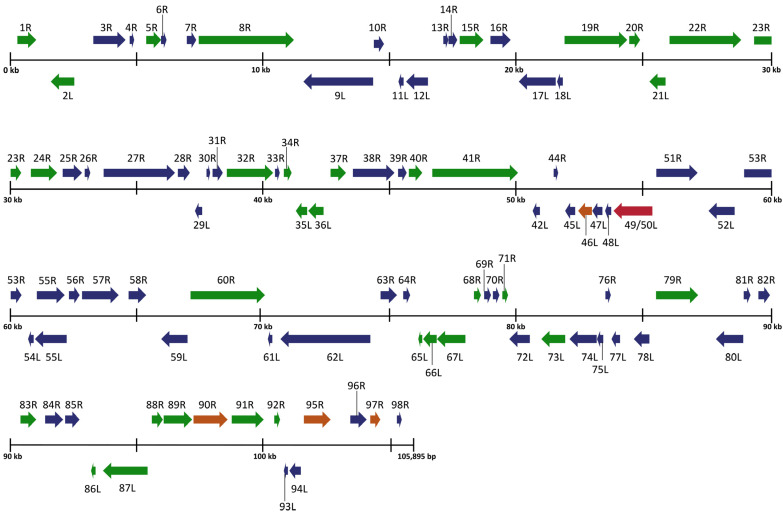
Schematic organization of the *Rana sylvatica* ranavirus (RSR) genome. ORFs are represented by arrows indicating approximate size and direction of transcription based on the position of the methionine initiation and termination codons. Genes represented in blue denote 100% amino acid identity between FV3 (AY548484.1), SSME (KJ175144.1), and RSR. Genes represented in green denote 100% amino acid identity between RSR and FV3, while genes represented in orange indicate 100% amino acid identity between RSR and SSME. Red denotes a gene unique to RSR genome.

To expand on the preliminary identification of ranavirus identified by Forzán et al. [27], we completed a full genomic analysis and found a unique FV3-like isolate that we designated *Rana sylvatica* ranavirus (RSR). Our sequence showed high similarity to both the published FV3 [34] and SSME [26] genomes with sequence identities of 99.98% and 99.64%, respectively. For comparison, FV3 has a genome length of 105,903 base pairs (bps) and is composed of 98 non-overlapping open reading frames (ORFs) [34], while SSME is composed of 105,070 bps with 95 predicted ORFs [26]. RSR is composed of 105,895 bps with a GC content of 55.1% and 97 predicted ORFs (Table 1). Despite the high overall sequence identity between FV3, SSME, and RSR, marked differences were evident in RSR in several regions of the genome (Figure 2).

### 3.2. Gene Variation between FV3, SSME, and RSR

**Figure 3:**
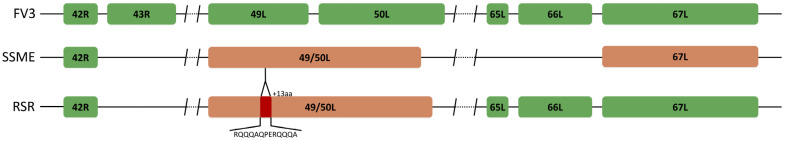
Unique ORF composition of *Rana sylvatica* ranavirus (RSR). Sequence alignment of ORFs 43R, 49L, 50L, 65L, and 66L in FV3 (AY548484.1), SSME (KJ175144.1), and RSR. RSR contains ORFs 65L and 66L; however, 43R is non-coding due to a frameshift mutation. RSR has the merger of ORFs 49L and 50L, designated 49/50L, like SSME, but contains a unique 13 amino acid insertion (red). Orange represents ORFs unique to the SSME genome.

An analysis of potential gene products revealed similarities between RSR and both FV3 and SSME; however, RSR possesses several ORFs that are unique to either FV3 or SSME (Figure 2). For example, RSR contains ORFs 46L, 90R, 95R, and 97R, which show 100% amino acid identity to their counterparts in the SSME genome (Figure 2); these ORFs differ from the FV3 genome through either (1) a single conservative mutation (95R and 97R), (2) a non-conservative missense mutation (90R), or (3) a frameshift mutation resulting in a gene elongation in SSME (46L) [26]. RSR contains the same frameshift mutation due to a single nucleotide deletion as SSME ORF 43R, resulting in the loss of the original stop codon likely rendering it non-coding (Figure 3) [26]. In addition, RSR contains ORF 49/50L, which is characteristic of SSME, but has a unique 13 amino acid insertion (Figure 3). Finally, FV3 is characterized by the presence of both ORF 65L and 66L [34], while SSME has a 757 base pair deletion resulting in the absence of ORF 65L and a non-functional 66L [26]. RSR lacks this base pair deletion and consequently contains the functioning ORFs 65L and 66L that are characteristic of FV3 (Figure 2 and Figure 3).

### 3.3. In Vitro Replication Variation between FV3, SSME, and RSR

**Figure 4:**
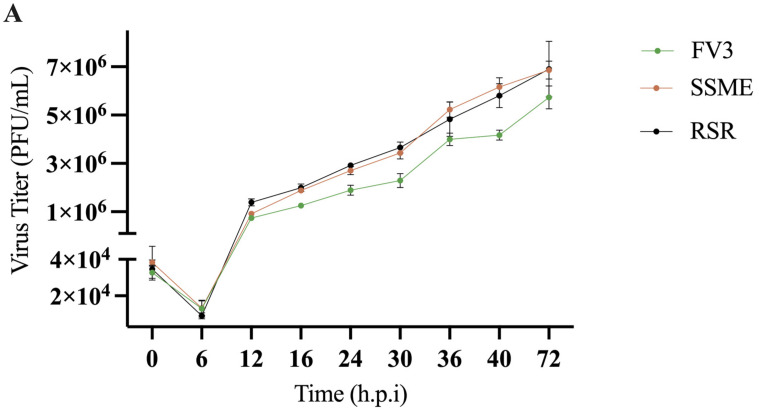

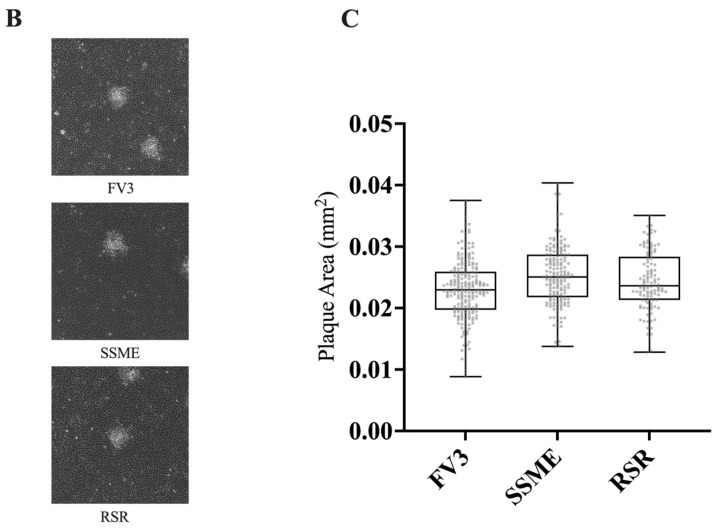
In vitro growth kinetics for FV3, SSME, and *Rana sylvatica* ranavirus (RSR) in EPC cells. (**A**) A single-step growth curve was performed with an equivalent number of cells and infected with FV3 (green), SSME (orange), or RSR (black) at an MOI of 5. Viruses were harvested at the indicated time points post-infection and quantified by plaque assay. Data are presented as mean (PFU/mL) ± SD. (**B**) Plaque area analysis was completed by exposing cells to FV3, SSME, or RSR for 4 h at an MOI of 0.1. After 4 h, inocula were removed and cells were overlaid with 0.75% methylcellulose, and images were taken 72 h post-infection. Images are representative of 3 independent experiments. (**C**) Plaque area (mm^2^) was quantified using Image J. Whiskers represent minimum and maximum values. No statistical significance was found using a Kruskal–Wallis test (*n* = 3).

In conjunction with genomic comparison, in vitro analysis was conducted to determine if the reported genetic variations would produce differences in viral replication. To evaluate the impact of genomic differences, a single-step growth curve was performed on FV3, SSME, and RSR at an MOI of 5 in EPC cells over a 72 h period. Growth kinetics for FV3, SSME, and RSR were similar until 16 h post-infection (h.p.i; Figure 4). At 16 h.p.i., RSR had comparable viral titers to SSME; however, RSR titers were 36.8% higher when compared to FV3 (Figure 4). At 72 h.p.i., viral replication was still comparable between RSR and SSME, but RSR produced viral titers that were 16.9% higher when compared to FV3 (Figure 4).

In addition to evaluating in vitro replication through a single-step growth curve, we also assessed plaque area. EPC cells were infected with FV3, SSME, or RSR at an MOI of 0.1 and plaque area was assessed after 72 h. RSR (M = 0.025 mm^2^) and SSME (M = 0.026 mm^2^) produced slightly larger plaques when compared to FV3 (M = 0.023 mm^2^), but this was not significant (*p* > 0.05; Figure 4).

## 4. Discussion

Ranaviruses are a group of globally emerging pathogens of economic and ecological significance. Ranaviruses are found on all continents except Antarctica and are known to infect fish, amphibians, and reptiles with the potential for interclass transmission [35,36]. In populations infected with ranaviruses, mass mortalities are routinely reported [6,9,11,13,17,37,38,39,40] and in some instances have resulted in host species extirpation [37,38]. Given their diverse host range and potential to reduce biodiversity, understanding the mechanisms behind ranavirus tropism and pathogenicity is important.

In the current study, we isolated a FV3-like ranavirus, designated *Rana sylvatica* ranavirus (RSR) from a die-off event in Wood Buffalo National Park, Canada. We were able to construct the complete genomic sequence of RSR and draw comparisons between two closely related isolates, FV3 and SSME. Comparisons revealed that RSR has ORFs 43R, 65L, and 66L (Figure 2 and Figure 3) which are unique to the FV3 genome [34]; these are non-coding regions in SSME [26]. ORF 43R is a 171 amino acid protein that contains a transmembrane domain [26,34,41], 65L is a 54 amino acid putative surface protein with a collagen triple helix (Table 1) [26,34,41], and 66L is a 183 amino acid proposed surface protein (Table 1) [26,34,41]. The most significant finding was that RSR has ORF 49/50L, which is consistent with SSME, but has a unique 13 amino acid insertion (Table 1; Figure 3). In SSME, 49/50L contains a SAP domain, a DNA/RNA-binding domain, and therefore may have a role in ranavirus replication [26].

The composition of RSR with regards to the presence of 43R, 65L, 66L, and 49/50L highlights the need for complete genomic sequencing. These genes fall outside of the 26 core genes in the *Iridoviridae* family. Therefore, if traditional methods relying on either partial or complete sequencing of the major capsid protein (MCP) and/or a subset of core genes were utilized, the unique composition of this virus isolate would remain unknown. Correspondingly, this study illustrates the need to continually report on ranaviruses isolated from wild populations. FV3 [34,42], SSME [26], and RSR are North American isolates that have significant sequence similarity but differ in original host species, genetic composition, and ability to induce mortality [26]. As such, ranaviral isolation from wild populations not only provides critical information regarding spatial and temporal distribution but it also highlights the genomic factors that may be contributing to disease dynamics.

In addition to the complete genomic sequencing of RSR, we further assessed in vitro viral growth kinetics. We found that, while there were differences in viral titers, there was no significant difference in plaque area for FV3, SSME, or RSR (Figure 4). Analysis of their genomes revealed that most genomic differences exist in immediate-early (43R, 66L) rather than late genes (65L) [41]. Similarly, FV3 ORFs 49L and 50L are characterized as immediate-early and delayed-early genes, respectively [41]; SSME ORF 49/50L has not be temporally classified. While the function of these proteins may be unknown, it has been postulated that immediate-early and delayed-early genes have roles in immune evasion and nucleic acid metabolism, while late genes may be involved in viral assembly [34]. Although the current study did not examine intergenic regions within RSR, intergenic regions in FV3 range in size from 20–900 nucleotides and account for 20% of the genome [43]. It has been proposed that these intergenic regions encode viral *cis*-regulatory element (v-CRE) mimics that interact with NF-kB, IFN, and STAT family members to circumvent cytokine and host antiviral immune responses [43]. Given the potential roles of the aforementioned genes and intergenic regions in immune regulation, it is plausible that RSR has adapted immune evasions strategies that cannot be fully ascertained in vitro. This thereby strengthens the need to complete virus challenge studies using RSR, SSME, and FV3 in a diverse group of hosts [44].

In summary, this study provided the complete genomic sequence of *Rana sylvatica* ranavirus (RSR) and found that it has a unique composition compared to two closely related isolates, SSME and FV3, thereby highlighting the importance of complete genomic sequencing. Moreover, this study assessed viral replication in vitro, and while we did not find any significant differences between isolates, we argue for the need to draw in vitro and in vivo comparisons between closely related isolates as this may help reveal key contributors to ranavirus host tropism and pathogenicity.

## Figures and Tables

**Table 1:** Description of nucleotide start/stop locations, amino acid length, along with predicted functions of ORFs in RSR.

ORF ^a^	Start/Stop (AA ^b^)	Predicted Function/Conserved Domain
1R	272–1042 (256)	Putative replicating factor, putative DNA packaging protein, poxvirus late transcription factor VLTF3–like domain
2L	1649–2611 (320)	Putative myristylated membrane, poxvirus entry-fusion-complex G9/A16 domain
3R	3418–4734 (438)	IIV-6 ORF 229L-like protein
4R	4775–4957 (60)	Hypothetical protein, transmembrane domain
5R	5390–6004 (204)	Similar to US22 herpesvirus early nuclear protein, FPV ORF 250–like protein
6R	6007–6234 (75)	Hypothetical surface protein
7R	7025–7411 (128)	Hypothetical surface protein
8R	7503–11,384 (1293)	DNA-dependent RNA polymerase II largest subunit
9L	11,753–14,599 (948)	Putative NTPase, N-terminal helicase domain of the DEAD-box helicase, helicase C-terminal domain
10R	14,615–15,028 (137)	Hypothetical protein, transmembrane domain
11L	15,376–15,588 (70)	Hypothetical protein, transmembrane domain
12L	15,654–16,547 (297)	Hypothetical surface protein
13R	17,088–17,294 (68)	Hypothetical surface protein
14R	17,309–17,668 (119)	Hypothetical surface protein
15R	17,764–18,732 (322)	AAA-ATPase, vaccinia virus A32-like protein
16R	19,012–19,839 (275)	Putative integrase-like protein
17L	20,080–21,588 (502)	Hypothetical surface protein
18L	21,626–21,862 (78)	Hypothetical protein, transmembrane domain
19R	21,914–24,469 (851)	Similar to LCDV1 ORF 10L uncharacterized conserved protein, putative serine/threonine-protein kinase, 2-cysteine adaptor domain, protein kinase-like catalytic domain
20R	24,517–24,963 (148)	Hypothetical protein, transmembrane domain
21L	25,200–25,859 (219)	Similar to ISKNV ORF 56L protein
22R	25,989–28,910 (973)	Putative D5 family NTPase/ATPase, C-terminal primase 2 domain
23R	29,288–30,436 (382)	Hypothetical surface protein
24R	30,819–31,916 (365)	Hypothetical protein
25R	32,110–32,898 (262)	p31K protein
26R	32,965–33,195 (76)	Truncated putative e1F-2alpha-like protein
27R	33,726–36,638 (970)	Putative tyrosine kinase, CAP10 putative lipopolysaccharide modifying enzyme, glycosyl transferase superfamily 90 domain
28R	36,687–37,175 (162)	Hypothetical protein, RNA polymerase Rpb5 C-terminal domain
29L	37,354–37,650 (98)	Hypothetical surface protein
30R	37,852–38,004 (58)	Hypothetical protein, transmembrane domain
31R	38,066–38,485 (139)	Hypothetical surface protein
32R	38,535–40,424 (629)	Neurofilament triplet H1-like protein
33R	40,507–40,698 (63)	Hypothetical protein, transmembrane domain
34R	40,842–41,162 (106)	Similar to human parainfluenza virus 1-like L-protein, seven-transmembrane G protein-coupled receptor superfamily domain
35L	41,254–41,715 (153)	Hypothetical protein
36L	41,728–42,351 (207)	Hypothetical protein
37R	42,747–43,376 (209)	Putative NIF/NLI interacting factor, HAD-like superfamily domain
38R	43,517–45,214 (565)	Hypothetical protein, ribonucleoside diphosphate reductase alpha subunit barrel domain
39R	45,320–45,670 (116)	Putative hydrolase of the metallo-beta-lactamase superfamily
40R	45,759–46,307 (182)	Hypothetical protein, transmembrane domain
41R	46,689–50,186 (1165)	Similar to RRV orf2 protein
42L	50,682–50,939 (85)	Hypothetical surface protein
44R	51,473–51,658 (61)	Hypothetical surface protein
45L	51,934–52,344 (136)	Similar to LCDV1 orf88, surface protein
46L	52,398–52,961 (187)	SSTIV orf049L-like protein, neurofilament triplet H1-like protein, microneme/rhoptry antigen
47L	53,086–53,502 (138)	Hypothetical surface protein
48L	53,505–53,756 (83)	Hypothetical surface protein
49/50L	53,865–55,451 (528)	LCDV1 orf58-like protein, SAP DNA binding domain, RGI orf50L-likeprotein, SSTIV orf052L-like protein
51R	55,531–57,216 (561)	Hypothetical surface protein
52L	57,473–58,540 (355)	Putative 3-beta-hydroxy-delta 5-C27 steroid oxidoreductase-like protein, Rossmann-fold NAD(P)-binding domain-containing protein
53R	58,878–60,446 (522)	Similar to LCDV1 orf20, L1R F9L superfamily domain, putative myristoylated protein, transmembrane domain
54L	60,661–60,891 (76)	Putative nuclear calmodulin-binding protein
55L	60,929–62,224 (431)	Helicase-like protein, N-terminal helicase domain of the DEAD-box helicase
55R	61,073–62,212 (379)	40 kDa hypothetical surface protein
56R	62,312–62,749 (145)	Hypothetical protein
57R	62,863–64,359 (498)	Putative phosphotransferase, serine/threonine protein kinase
58R	64,684–65,397 (237)	Hypothetical surface protein
59L	65,948–67,006 (352)	RGV 9807-like protein, hypothetical surface protein
60R	67,168–70,209 (1013)	DNA polymerase-like protein
61L	70,218–70,400 (60)	Hypothetical surface protein
62L	70,843–74,508 (1221)	DNA-dependent RNA polymerase II second largest subunit domain
63R	74,887–75,381 (164)	Putative dUTPase-like protein
64R	75,521–75,808 (95)	Putative interleukin-1 beta convertase precursor, caspase recruitment domain/DEATH
65L	76,201–76,365 (54)	Hypothetical protein, collagen triple helix repeat
66L	76,362–76,913 (183)	Hypothetical protein
67L	76,968–78,131 (387)	Ribonucleoside diphosphate reductase beta subunit-like protein
68R	78,414–78,701 (95)	Hypothetical protein
69R	78,837–79,103 (88)	Hypothetical protein, transmembrane domain
70R	79,121–79,495 (124)	Hypothetical surface protein
71R	79,535–79,768 (77)	Hypothetical protein
72L	79,825–80,541 (238)	Hypothetical surface protein
73L	80,989–81,963 (324)	Putative NTPase/helicase-like protein
74L	82,138–83,250 (370)	Hypothetical surface protein
75L	83,282–83,536 (84)	Putative LITAF/PIG7 possible membrane associated motif in LPS-induced tumor necrosis factor alpha factor, transmembrane domain
76R	83,599–83,820 (73)	Hypothetical protein
77L	83,817–84,164 (115)	LCDV1 orf2-like protein, hypothetical surface protein
78L	84,749–85,387 (212)	Hypothetical protein, RNA recognition motif (RRM) superfamily
79R	85,523–87,241 (572)	Putative ATPase-dependent protease
80L	87,864–88,979 (371)	Ribonuclease-like protein, RIBOc domain-containing protein
81R	89,035–89,313 (92)	Transcription elongation factor SII, zinc ribbon domain-containing protein
82R	89,442–89,915 (157)	Immediate early protein ICP-18
83R	90,365–91,009 (214)	Cytosine DNA methyltransferase
84R	91,381–92,118 (245)	LCDV1-like proliferating cell nuclear antigen
85R	92,193–92,780 (195)	Putative deoxynucleoside kinase
86L	93,170–93,355 (61)	Hypothetical protein
87L	93,708–95,525 (605)	Hypothetical protein
88R	95,558–96,010 (150)	Putative Evrl-air-augmenter of liver regeneration; Erv1/Alr family FAD-linked sulfhydryl oxidase
89R	96,078–97,244 (388)	Hypothetical protein
90R	97,337–98,728 (463)	Major capsid protein
91R	98,852–100,039 (395)	Immediate early protein ICP-46
92R	100,390–100,629 (79)	Hypothetical protein
93L	100,811–100,978 (55)	Hypothetical protein
94L	101,088–101,555 (155)	Similar to Regina ranavirus P8.141C protein; PDDEXK family nuclease domain
95R	101,648–102,739 (363)	Putative DNA repair protein RAD2, PIN (PilT N-terminus) domain, Xeroderma pigmentosum G N-region, helix-hairpin-helix
96R	103,541–104,212 (223)	Hypothetical surface protein
97R	104,295–104,708 (137)	Putative myeloid cell leukemia protein; transmembrane domain; MCL–1 region
98R	105,474–105,674 (67)	Hypothetical protein

^a^ Direction of transcription is indicated by left “L” for anti-sense strand and right “R” for sense strand of DNA. ^b^ AA- number of amino acids of each putative protein.

## Data Availability

All data generated or analyzed during this study are either incorporated within this published article or accessible upon reasonable request to the corresponding author. The FV3 (AY548484.1) and SSME (KJ175144.1) reference genomes are freely available from GeneBank, and RSR has been assigned Genebank accession number PQ109930.
